# Ring-Pins combined with cable cerclage for the fixation of displaced inferior patellar pole fractures

**DOI:** 10.3389/fsurg.2022.1043822

**Published:** 2023-01-16

**Authors:** Zhen Jian, Jianbo Jia, Langqing Zeng, Dejian Li, Xu Zhang, Jianhua Zhou, Chengqing Yi, Baoqing Yu, Rongguang Ao

**Affiliations:** ^1^ Department of Orthopedics, Shanghai Pudong Hospital, Fudan University Pudong Medical Center, Shanghai, China; ^2^ Department of Orthopedics, Shanghai East Hospital, Tongji University School of Medicine, Shanghai, China; ^3^ Department of Orthopedics, Zhuhai People's Hospital, Zhuhai Hospital Affliated with Jinan University, Zhuhai, China; ^4^ Department of Orthopedics, Shanghai Pudong New Area People's Hospital, Shanghai, China

**Keywords:** ring-Pin, inferior patellar pole, fixation, cable cerclage, fracture

## Abstract

**Objective:**

The study aimed to present the clinical results and complication rates of ring-pins with cable cerclage for treating the inferior pole of patella fracture.

**Method:**

A study that retrospectively reviewed consecutive patients of the displaced inferior pole of patella fracture (AO/OTA 34-A1) operated with a ring-pin tension band using cable cerclage between October 2015 and October 2017 was performed. The duration of surgery, motion range of the knee, function outcomes, and complications were recorded.

**Results:**

The average follow-up of 31 patients was 21 months. The mean operation time was 50 min. Fractures in all 31 patients healed at a mean duration of 8 weeks. There was no infection, no withdrawing of ring-pins, no implant breakage, and no loss of fracture reduction. The mean range of motion was 120°, and no patient complained of implant irritation at the final follow-up. The average Bostman score was 29.0 points, and 28 patients graded clinical outcomes excellent and 3 patients graded clinical outcomes good at the last follow-up.

**Conclusions:**

Ring-pin combined with cable cerclage for treating the displaced inferior pole of patellar fracture is simple, and the postoperative internal fixation-related complication rate is low. It is a good choice for treating the displaced inferior pole of the patellar fracture.

## Introduction

The inferior pole of patella fracture is not very common and accounts for 5% of all patellar fractures (1, 2). The inferior patella pole fracture is usually small comminuted fragments and is always associated with a rupture of the extensor mechanism of the knee. The aim of the operation is to restore the continuity of the extensor mechanism by fixing the fracture fragment and to allow early movement of the knee.

The most widely adopted method for treating displaced patellar fractures is to operate with internal fixation using a tension band (3, 4), which could transform the anterior tension force into compressive force on the posterior surface of the patella. The tension band techniques provide good fracture healing and functional recovery. Xu et-al. (5) reported that the modified tension band technique combined Kirschner wires (K-wires) with cable cerclage for the displaced inferior pole of patella fracture to provide firm fixation with satisfying clinical outcomes in knee function. However, K-wires are usually associated with possible loss of reduction and irritation of the bursa suprapatellaris and skin due to migration. Patients with symptomatic hardware often have to receive secondary operation to remove the fixation.

Ring-pins may avoid such problems. It was reported that the ring-pin fixation was first developed in 2000 by Nakashima Medical Co. Ltd. (Okayama, Japan) (6, 7). The special pin is composed of three parts: a sharp wire in the distal part, a ring-end as the connection section, and a proximal part for securing the grip over the power tool. The operators can fold the proximal section above the ring-end and remove the part after inserting the pin into the bone correctly. The ring was used across the cable, which benefits fraping the cable and compressing the fractures. What is more, the ring-pins were scarcely possible to withdrawing because they were fixed by the cable through the ring. Theoretically speaking, the ring-pin combined with cable cerclage would avoid withdrawing the K-wire, providing more stable fixation compared with K-wires. The objective of the retrospective study was to present the clinical results and complication rates of ring-pins with cable cerclage for treating the inferior pole of patella fracture.

### Patients and methods

A retrospective study that reviewed consecutive patients of the displaced inferior pole of patella fracture (AO/OTA 34-A1) operated with a ring-pin tension band using cable cerclage between October 2015 and October 2017 was performed. Institutional review board approval for the study was obtained. All surgeries were operated by orthopedic surgeons with the same experience and qualifications.

Thirty-six patients suffering from displaced inferior patellar pole fractures and treated with ring-pins combined with cable cerclage were chosen as candidate participants. The medical records of all participants were reviewed. Exclusion criteria were the loss of follow-up within 1 year after surgery (*n* = 1), the presence of concomitant fractures (*n* = 1), and the presence of open fractures (*n* = 3). Therefore, the study comprised 31 patients who sustained displaced inferior patellar pole fractures and underwent open reduction and internal fixation with ring-pin tension bands with cable cerclage. The average age of the patients was 56 years (range 32–78 years). In our study, 13 patients were men and 18 were women. The average duration from surgery to initial injury was 3 days (range 1–4 days).

All patients in our department were asked to follow up monthly after surgery until fracture union. The duration of surgery during the procedure was recorded. The Bostman score was used to assess functional recovery postoperation. The overall score is 30 points: 28–30 points meant excellent; 20–27 points meant good; and less than 20 points meant poor (8). Postoperative complications that were documented included infection, nonunion, implant breakage, ring-pin withdrawal, and implant irritation.

### Surgical techniques

**Figure 1:**
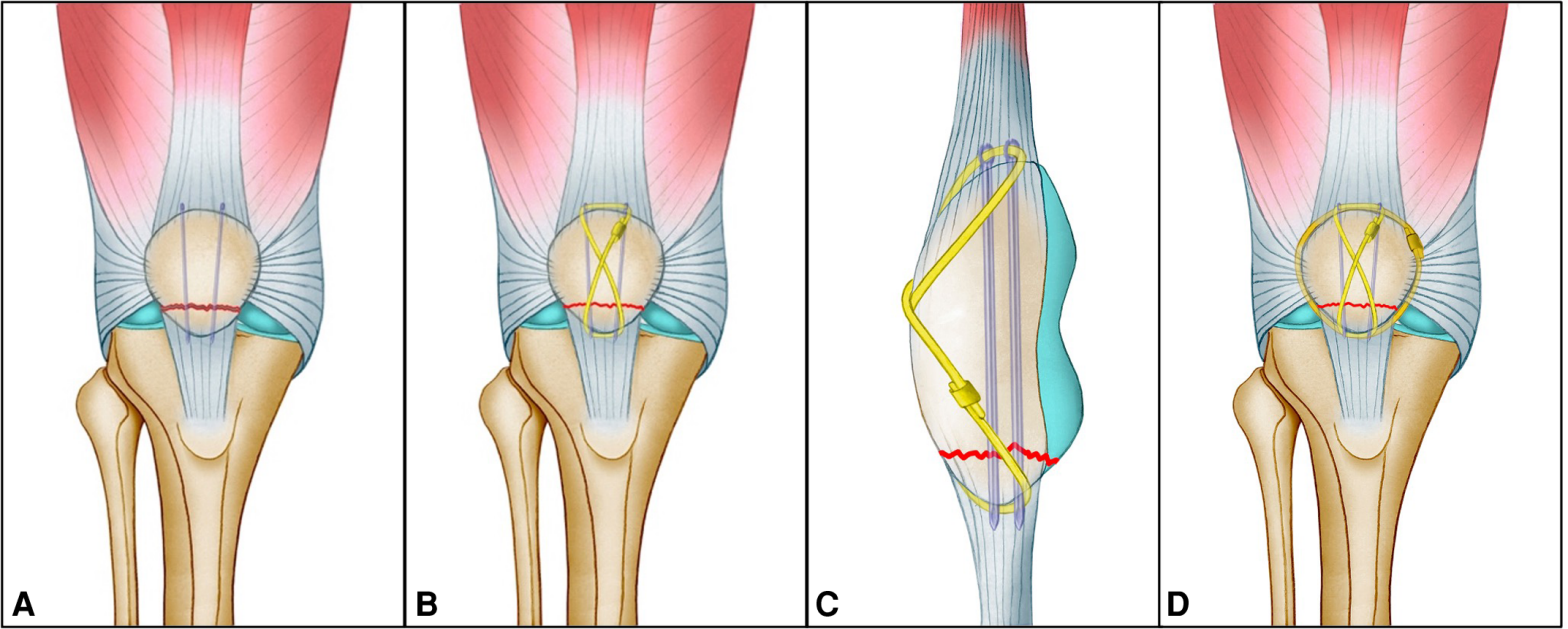
Diagrams showing that the displaced inferior patellar pole fracture was reduced and fixed by a ring-pin combined with cable cerclage. (**A**) Main fragment of the inferior pole threaded by the two ring-pins. (**B**) Anteroposterior and (**C**) lateral views showing insertion of the cable over the front of the patella to form a figure-8. (**D**) Final fixation of the inferior patellar pole fracture.

The patient was placed in a supine position after anesthesia, and a pneumatic tourniquet was conventionally applied. A midline incision from the proximal patella to the tibial tubercle was made vertically over the knee, and full-thickness lateral and medial skin flaps were developed. The inferior pole patella fragment was exposed, and soft tissue detached from the fragment was preserved. Hematoma and soft tissue from the fracture and knee joint were removed. After full extension of the knee, the fracture was reduced with two reduction forceps with gentle force. Two 1.6-mm ring-pins were inserted from the proximal to the inferior pole in an axial direction. The main fragment of the inferior pole should be threaded by the two ring-pins (Figure 1A). The configuration of the pins could be either parallel or nonparallel according to the size of the main fragment of the inferior pole. The parallel configuration of pins was commonly used for noncomminuted inferior patellar pole fractures; the nonparallel configuration of pins, which was slightly wide at the top and slightly narrow at the inferior, was usually used for comminuted inferior patellar pole fractures because of the smaller size of the main fragment. Then, anteroposterior and lateral view fluoroscopy was applied to confirm the position of the ring-pin and the reduction of fracture. One 1.3-mm-diameter titanium cable (Zimmer Inc., USA) was passed through the rings, followed by knocking on the ring-pin end closed to the proximal patella bone to avoid irritation of the suprapatellar bursa. It was ensured that the titanium cable could slide through the rings. Then, the cable was crossed over the front of the patella to form a figure-8 (Figure 1B,C). A tension lever was tightened up and locked. To stabilize the fracture further, the surgeons could insert another titanium cable close to the edge of the patella, and the cerclage cable was tightened by the tension lever (Figure 1D). The proximal parts of the ring pins were removed. The distal ring pings were cut as close to the osseous border of the inferior patellar pole as possible to avoid irritating the skin.

The number-0 absorbable sutures were routinely used to repair the retinaculum and ligamentum patellae. Before operations were completed, the surgeons fully flexed the knee to 130° to test the stability of internal fixation. Fluoroscopy was applied to confirm the reduction and fixation before closuring the wound. Standard wound closure was applied layer by layer using number-2 absorbable sutures for the subcutaneous tissue and a subcuticular stitch for the skin.

### Postoperative rehabilitation

The knee was placed in a hinged knee brace. All patients were allowed to initiate muscle strengthening exercises postoperation and passive flexion of the knee 2–3 days after surgery. Partial weight-bearing began 2–3 weeks after surgery and gradually went to full weight-bearing by 4–6 weeks. The range of flexion motion of the knee was also increased gradually. Stages of active knee flexion started at 30°, then expanded to 60° in 2–4 weeks, and changed to 90° in 4–6 weeks.

## Results

**Figure 2:**
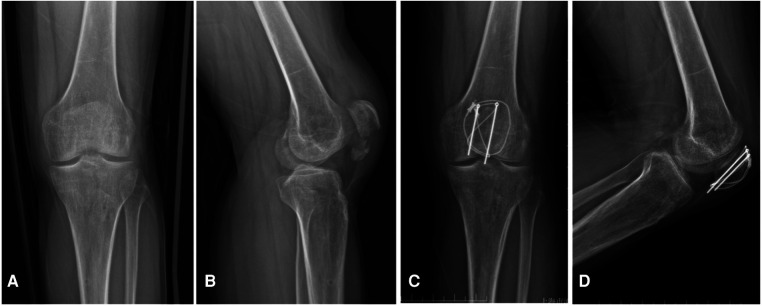
Case example of displaced inferior patellar pole fracture (Male, 36 years, left knee) using the ring-pin combined with cable cerclage. Preoperative radiographs in anteroposterior (**A**) and lateral (**B**) views showing a displaced inferior patellar pole fracture. Radiographs in anteroposterior (**C**) and lateral (**D**) views at 3 months follow-up, which show that the fracture was healed and the implants were in position without failure.

The average follow-up duration of the 31 patients was 21 months (18–35 months). The average operation duration was 50 min (40 to 75 min). The fractures healed at an average time of 8 weeks (7–11 weeks). A typical case is shown in Figure 2.

There was no infection, no withdrawing of ring-pins, no implant breakage, and no loss of fracture reduction. At the final follow-up, no patient complained of irritation of internal fixation. The mean range of motion was 120° (115°–130°). The average Bostman score at the final follow-up was 29.0 points (26.5–30). Additionally, 28 patients graded clinical outcomes excellent and 3 patients graded clinical outcomes good.

### Discussion

Inferior patellar pole fractures are a distinct group of completely extra-articular avulsion injuries, usually measuring less than 15 mm in vertical length (2, 9). The key point of operation is to effectively fix the main fragment of the inferior pole fracture to allow early movement of the knee. Treatment for displaced inferior pole of patella fractures is challenging because it is usually with small fragments and too comminuted to fix and maintain reduction.

Several surgical methods are used for the treatment of the displaced inferior pole of patella fracture, including separate vertical wiring, rim-plate-augmented separate vertical wiring (2), fixed with a basket plate (10) or augmented with Krachow sutures (11), K-wires combined with cable cerclage (5), or anchor suture fixation (12). However, there are no widely accepted methods that have been identified for treating displaced inferior patellar pole fractures until now.

Separate vertical wiring is easy to perform with satisfactory clinical results. Kim et al. (13) reported 18 patients who received separate vertical wirings for the extra-articular fracture of the inferior patella pole. The ultimate mean flexion motion of the knee was 127.6°, and the mean Bostman score was 27.5 points. A biomechanical study (2) confirmed that the ultimate load to failure when using separate vertical wiring was 250 N and the stiffness was 280 N/mm, which was not strong enough to allow carrying out knee joint function exercises gradually under the extra protection of braces after surgery.

The final failure load of the basket plate for the fixation of the distal patella pole was the highest, achieving 421.66 ± 45.90 N (2). Matejcic et al. (14) reported that passive activities could be started on the first day after surgery in patients with basket plates. However, injury to the patellar tendon and the relative bulk of the plate with the thin, soft tissue layer over the anterior patella are the main drawbacks of the basket plate (15). The biomechanical study by Krkovic et al. (16) found that the basket plate was bond to cause significant shortening and rupture of the patellar tendon. Furthermore, this specific plate is not available in all institutions (15).

Anchor suture fixation has the advantages of definite clinical effects and avoiding secondary removal (12). Sixty patients with distal pole patellar fracture were retrospectively studied by Kadar et al. (17), and they found that the anchor suturing technique was not inferior compared with partial patellectomy for pain after displaced inferior pole of patella fracture and functional recovery and was superior with regard to operative time. The reoperation rate of 14.8% was also recorded in the anchor suture group. The most frequent complications of the technique are infections and implant failure. To ensure the fixation strength, it is usually recommended to fix the knee with plaster or brace in an extended position for 4–6 weeks after surgery (17, 18). Long-term embolization may lead to knee joint adhesion, quadriceps muscle atrophy, deep vein thrombosis, and limited knee flexion function.

Ideal operation methods should be sufficient fixation stability, a relatively simple surgical procedure, and early motion of the injured knee. K-wire fixation with tension band wiring is still the most frequent method to treat transverse patellar fractures, and it can provide sufficient fixation stability according to the theory of tension band (3, 9). However, there is an inherent disadvantage for conventional K-wires, such as migration and breakage, with the subsequent painful and prominent implant in the knee. Lazaro et al. (19) claimed a rate of 37% hardware removal due to symptomatic and prominent hardware as a result of continuous soft tissue irritation or fixation breakage. Hung et al. (20) reported that the rate of complications associated with stainless steel wire loops was 47% and 15% of patients had related symptoms that needed wire removal. Meanwhile, it is more difficult to fix the inferior patellar pole fracture than transverse patellar fractures because the inherent weakness of the inferior pole fragments and the small size of the fracture fragments prevent sufficient stabilization by common wiring.

Kirschner wires combined with cable cerclage may still be a good choice. The modified tension band technique connected Kirschner wires with cable cerclage using a Cable Grip System and can provide an effective alternative to reduce the rate of hardware loosening, wire breakage, and soft tissue irritation caused by stainless steel wires (5). Compared to stainless steel wires, titanium cables can provide more reliable stability and decrease complications such as loosening and breakage. Stainless steel wires are easy to break and loosen in fixation that usually lead to a high incidence of prominent hardware, accounting for almost 50% of all risk of secondary surgical removal of tension band fixation (21, 22).

Ring-pins are designed to lock the pin with the cable through the ring-end that is bonded to prevent postoperative migration (7). A large cohort of 334 comminuted patellar fracture cases reported by Zhu et al. (6) demonstrated that ring-pin tension band fixation is superior to cannulated-screw tension band and Kirschner wire tension band fixation due to a lower rate of hardware failure and secondary removal surgery, respectively. In our study, there was also no implant failure or loss of fracture reduction. At the last follow-up, no patient complained of uncomfortableness due to implant irritation. At the final follow-up, 28 patients graded clinical outcomes excellent and three patients graded clinical outcomes good according to the Bostman score.

## Conclusions

Ring-pins combined with cable cerclage for treating displaced inferior pole fractures of the patella are simple and facilitate early functional exercise due to firm fixation. In addition, the postoperative internal fixation-related complication rate is low. In conclusion, it is a good choice for treating displaced inferior patellar pole fractures.

## Data Availability

The raw data supporting the conclusions of this article will be made available by the authors without undue reservation.
